# Outpatient Parenteral Antimicrobial Therapy Outcomes Metrics Assessment Survey

**DOI:** 10.1093/ofid/ofaf283

**Published:** 2025-05-12

**Authors:** Jina Makadia, Amber C Streifel, Cara D Varley

**Affiliations:** Division of Infectious Diseases, Department of Medicine, Oregon Health & Science University, Portland, Oregon, USA; Department of Pharmacy, Oregon Health & Science University, Portland, Oregon, USA; Department of Pharmacy, Oregon Health & Science University, Portland, Oregon, USA; Epidemiology Programs, School of Public Health, Oregon Health & Science University and Portland State University, Portland, Oregon, USA

**Keywords:** outcomes research, outpatient parenteral antimicrobial therapy, outcome metrics survey, perceived importance in OPAT, OPAT data tracking

## Abstract

We surveyed outpatient parenteral antimicrobial therapy team members to assess the importance of specific program metrics while eliciting collection barriers, which can guide establishing or restructuring programs. Several metrics were felt to be highly important but were not being collected by programs. Identified barriers included a lack of funding, technology support, and staffing.

## Teaser

We present the analysis of a cross-sectional, electronic survey of OPAT team members to assess program outcome metrics currently being used, the perceived importance of selected metrics, and barriers to collection. While a number of metrics were considered highly important, only a few programs were able to collect the data needed to track these metrics. Our data can provide guidance and support for establishing or restructuring OPAT programs to determine which outcome metrics to collect and track at a program level.

Outpatient parenteral antimicrobial therapy (OPAT) is integral to the care of patients receiving intravenous antimicrobials outside the hospital [1, 2]. OPAT has been shown to improve patient satisfaction and reduce length of hospital stay, hospital-acquired infections, and overall health care cost [2–5]. While many institutions have instituted formal OPAT programs, there are limited data on metrics or outcomes assessment related to OPAT programs. These data are critical for assessing the impact of quality improvement efforts and programmatic changes on the functionality and quality of OPAT programs.

## METHODS

We conducted a cross-sectional electronic survey of OPAT team members to assess program outcome metrics currently being used and the perceived importance of selected metrics (Supplementary Appendix 1). We collected respondent demographics, role in OPAT, and factors related to OPAT program structure. Participants rated OPAT metrics by perceived importance from 1 to 10, with 1 defining least important and 10 defining most important. Outcome metrics proposed in the survey were collected from a literature review, in addition to metrics planned for collection in our OPAT program [1–10].

We administered the anonymous survey via Qualtrics (https://www.qualtrics.com). We posted the survey link on the Infectious Diseases Society of America IDea Exchange and emailed it to a group of OPAT team members via an online OPAT chat group, both of which include members in the United States and internationally with a variety of clinical roles (providers, nurses, pharmacists). The survey link was open for data collection from 16 May to 16 August 2024, with 2 reminders posted.

We performed all analyses in SAS statistical software 9.4 (SAS Institute; https://www.sas.com). We used descriptive statistics to examine the perceived importance of OPAT metrics, as well as participant, institutional, and program characteristics, reporting proportions for categorical variables and median values (IQR, SD) for continuous variables.

## RESULTS

The OPAT survey was started by 112 participants; 87 (77.6%) scored at least 1 outcome metric and were included in analyses (Table 1). Among these 87 participants, 81 (92.9%) reported practicing in the United States. Most commonly, participants practiced in academic medical centers (n = 63, 72.4%) and were currently practicing in a formal OPAT capacity (n = 56, 64.4%).

**Table 1.** Survey Participant and OPAT Program Characteristics and Outcome Metrics Barriers (N = 87)

	No. (%)
Survey participant and OPAT program characteristics	
Gender: women	53 (60.9)
Race and ethnicity	
White	69 (79.3)
Asian	13 (15)
Combined group^a^	4 (4.6)
Country: United States	81 (92.9)
Institution setting	
Academic medical center	63 (72.4)
Community teaching medical center	13 (14.9)
Community nonteaching medical center	5 (5.7)
Private practice	3 (3.4)
Other^b^	7 (8.0)
Participant's role in OPAT	
Director/codirector	31 (35.6)
Member of the OPAT team: responsible for day-to-day program operation, involved with improvement processes	25 (28.7)
Provider who cares for patients with OPAT: involved with process improvement but not in the day-to-day logistics of OPAT program	18 (20.7)
Provider who cares for patients with OPAT: no involvement with program or process changes	9 (10.3)
Former OPAT member	2 (2.3)
Not involved in OPAT	2 (2.3)
Duration of participant's current role in OPAT, y	
<1	4 (4.6)
1–4	33 (37.9)
5–9	29 (33.3)
>10	19 (21.8)
Roles included in participant's OPAT model	
Physician	70 (80.5)
Advanced practice provider	25 (28.7)
Pharmacist	33 (37.9)
Nurse (BSN/RN)	37 (42.5)
Medical assistant	11 (12.6)
Social worker/case manager	5 (5.7)
Administration/nonclinical personnel	10 (11.6)
Participants with an FTE-supported role	39 (44.8)
Percentage of FTE support in those with an FTE-supported role	
<10	5 (5.7)
10–25	10 (11.5)
25–50	12 (13.8)
50–75	6 (6.9)
>75	6 (6.9)
Percentage of weekly hours spent on OPAT	
<10	25 (28.7)
10–25	30 (34.5)
25–50	16 (18.4)
50–75	5 (5.7)
>75	11 (12.6)
OPAT program includes patients with substance use disorders	62 (71.3)
Addiction medicine consult service available	47 (54.0)
Multidisciplinary discharge planning meeting	18 (20.7)
Antimicrobial stewardship program in OPAT	49 (56.3)
Required OPAT data reporting to hospital leadership	
Yes	13 (14.9)
Yes, but not regularly	14 (16.1)
Barriers to OPAT outcome metric data collection	
Staffing shortages	27 (31.0)
Lack of administrative support	28 (32.2)
Lack of funding	25 (28.7)
Lack of a data system to track patients or technology support to build a system	35 (40.2)
Lack of skilled nursing facility support	5 (5.7)
Lack of addiction medicine team	6 (6.9)
Other	6 (6.9)

Abbreviations: FTE, full-time equivalent; OPAT, outpatient parenteral antimicrobial therapy.

^a^Combined group: Hispanic/Latinx, Middle Eastern, North African, Black or African American, or those who declined response, due to counts <5.

^b^Other health care setting: Veterans Health Administration, not for profit health plan.

Of the 39 participants with full-time equivalent (FTE) support for OPAT, 24 (61.5%) reported weekly time spent equivalent to their FTE, and 10 (25.6%) and 5 (12.8%) indicated working more and less than their FTE, respectively. Of note, half of participants had an active current OPAT role (n = 45, 51.7%) without FTE support.

The inclusion of patients with substance use disorder in OPAT programs was reported by 62 (71.3%) participants; incorporation of antimicrobial stewardship (ASP) was noted by 49 (56.3%). Most participants (n = 49, 56.3%) were not required to report OPAT program data to hospital leadership.

**Figure 1.**
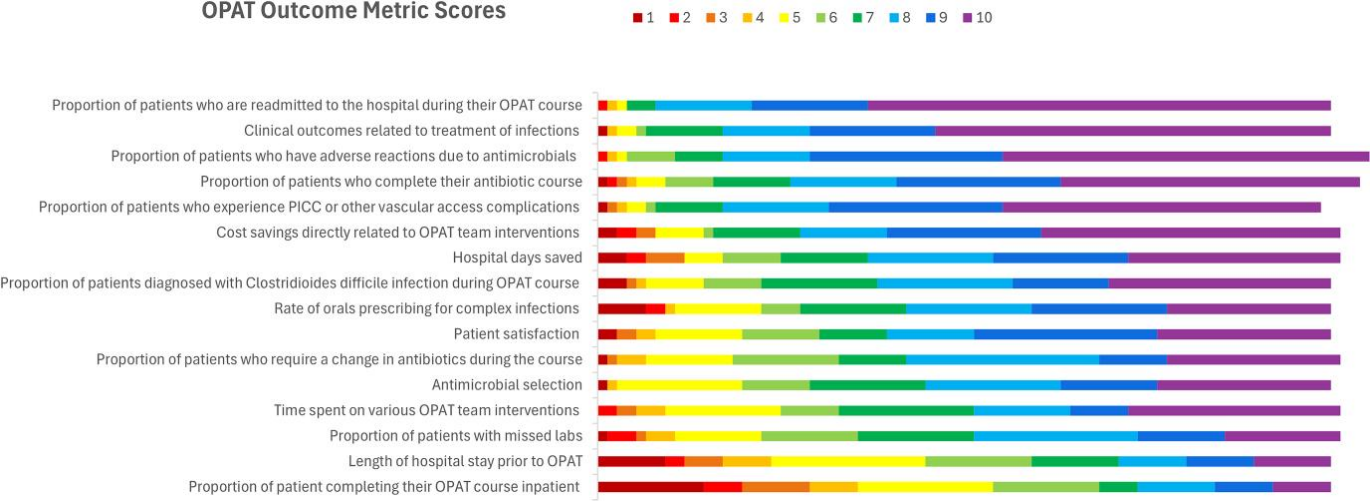
Distribution of OPAT outcome metric scores: 1 (least important) through 10 (most important). Abbreviations: OPAT, outpatient parenteral antimicrobial therapy; PICC, peripherally inserted central catheter.

A median score of 8 to 10 was seen in 12 of the 16 outcome metrics (Supplementary Table 1, Figure 1). The highest-scoring metrics included the proportion of patients with adverse reactions due to antimicrobials (10; SD, 1.39), the proportion of patients readmitted to the hospital during their OPAT course related to infection or antimicrobial adverse effects (10; SD, 1.45), and clinical outcomes (10; SD, 1.68). The lowest-scoring metrics included the proportion of patients completing their antibiotic course as inpatients (5; SD, 2.79) and length of hospital stay prior to OPAT (6; SD, 2.60).

Sixteen (18.4%) respondents listed additional outcomes that they were collecting: patient follow-up, proportion of patients enrolled in OPAT, dose adjustments, medication errors, rate of discharge plan verification, central line removal confirmation, and reasons for extension therapy.

The most commonly reported barriers to collecting metrics were lack of a data system to track patients or technology support to build a system (n = 35, 40.2%), followed by a lack of administrative support (n = 28, 33.2%), staffing shortages (n = 27, 31.0%), and lack of funding (n = 25, 28.7%).

## DISCUSSION

Although key components of OPAT programs have been proposed, significant variability exists among programs and within published literature, limiting comparison of metrics across programs and studies, especially internationally [6–8]. Our data provide metric selection guidance for establishing or restructuring OPAT programs and elicit barriers to successfully using those metrics in practice.

Survey responses indicate that metrics such as readmission rates, adverse reactions to antimicrobials, and clinical outcomes of treatment were felt to be highly important outcome metrics for OPAT programs. However, only a fraction of respondents reported that these data were being actively collected (37.9%, 27.6%, and 19.5% of responses, respectively), with respondents reporting multiple barriers to collection.

Many studies reporting clinical outcomes in OPAT have been published [9, 10]; however, per our survey, consistent collection of these metrics is limited. Clinical outcomes are of interest to an OPAT program to implement interventions around antimicrobial selection, dosing, and management of adverse effects. Yet, there are many other factors influencing these outcomes that must be considered and are outside the influence of an OPAT program, including comorbidities, adequate source control, retained hardware, antimicrobial resistance, and, especially in the United States, access to medication and adequate insurance coverage. More than half of survey respondents reported a formal ASP component. ASP efforts in OPAT may include antimicrobial selection, duration of therapy, and appropriate intravenous-to-oral switches. In the United States, antimicrobial selection is heavily influenced by insurance coverage and treatment setting and so requires different approaches than inpatient administration. However, tracking these data within an OPAT program may still shed light on opportunities for improvement [11–13].

Readmission rates were the most common metric collected by survey participants and are a common outcome reported in current OPAT literature [14–16]. The ability to capture readmission data has allowed us to identify risk factors for readmission specific to our OPAT population [14, 15]. While readmissions may be directly related to clinical outcomes and antibiotic-related adverse effects, the ability of an OPAT program to show cost savings is often tied to metrics such as readmission rate and reduced length of hospital stay [16]. Furthermore, demonstrating reduction in readmissions, adverse effects, and vascular line complications has additional benefit toward justifying a program's cost to hospital leadership. Readmissions during an OPAT course are multifaceted, however, and improvement in rates with various OPAT interventions has been shown to plateau, as there are indications for readmission outside the control of an OPAT team [3, 17, 18].

Measuring patient satisfaction in OPAT was felt to be important, although <2% of respondents reported collecting these data. Studies of patient satisfaction have been published [19–21], specifically related to preferences for antibiotics or infusion methods [22, 23], and ongoing evaluation may highlight real-time issues experienced and allow better coordination and quality improvement measures.

The most common barrier to collecting data was the lack of a system to track patients or technology support to build a tracking system. These are similar to barriers seen in OPAT programs in general [24]. It could be argued that dedicating more resources to efficient tracking and data collection systems could improve the overall efficiency of programs and thus warrants further investigation. The next most common was a lack of administrative and staffing support. There is a significant discrepancy in the FTE dedicated to OPAT and the actual time spent on OPAT tasks as reported by respondents. Similar discrepancies have been noted between time spent on OPAT and billable time [25], further illustrating the need for dedicated resources to follow metrics and implement changes to improve patient care. Based on our survey responses, programs have significant variability in terms of team makeup, addiction medicine consult, discharge planning, and ASP component, highlighting the need for standardized metric reporting to evaluate the impact of these components. An OPAT data registry in the United Kingdom has shown that standardizing metrics allows for compilation of data, allowing conclusions to be drawn about the efficacy and safety of OPAT programs [26]. Similar models in the United States and internationally would greatly enhance our ability to compile and use OPAT data and compare intervention impacts across programs to ultimately improve patient care.

Our study has limitations. The majority of respondents were practicing in academic centers within the United States, limiting generalizability to other settings. Additionally, OPAT team members not participating in the OPAT group chat or regularly reading the Infectious Diseases Society of America IDea Exchange would not be included, limiting program representation. To protect participant confidentiality, we did not collect facility data nor limit the number of respondents from each program, which limits our ability to determine program representation.

## CONCLUSION

While OPAT members reported a number of metrics highly important, few programs are able to collect the data needed to track these metrics. Efforts are needed to standardize program metrics and electronic health records for improved access to crucial OPAT data. These data can provide guidance for establishing or restructuring OPAT programs on metric selection.

## Supplementary Material

ofaf283_Supplementary_Data

## Supplementary Data

Supplementary materials are available at *Open Forum Infectious Diseases* online. Consisting of data provided by the authors to benefit the reader, the posted materials are not copyedited and are the sole responsibility of the authors, so questions or comments should be addressed to the corresponding author.

## Notes

***Author contributions*.** J. M.: investigation, methodology, writing–original draft, writing–review and editing. A. C. S.: conceptualization, investigation, methodology, data curation, writing–original draft, writing–review and editing. C. D. V.: conceptualization, investigation, methodology, formal analysis, data curation, visualization, writing–original draft, writing–review and editing, supervision.

***Survey participant consent*.** This study was reviewed and approved by the institutional review board at Oregon Health & Science University (IRB00027180). Per guidelines for anonymous surveys with minimal risk, an institutional review board–approved information sheet was provided to potential participants prior to beginning the survey, and consent to participate was implied with survey completion.

***Potential conflicts of interest.*** All authors: No reported conflicts.

## References

[1] Halilovic J, Christensen CL, Nguyen HH. Managing an outpatient parenteral antibiotic therapy team: challenges and solutions. Ther Clin Risk Manag 2014; 10:459–65. 24971015 10.2147/TCRM.S48906 PMC4069209

[2] Schmidt-Hellerau K, Baade N, Günther M, et al Outpatient parenteral antimicrobial therapy (OPAT) in Germany: insights and clinical outcomes from the K-APAT cohort study. Infection 2024; 52:1407–14. 38478255 10.1007/s15010-024-02199-9 PMC11289149

[3] Bouzigard R, Arnold M, Msibi SS, et al Outpatient parenteral antimicrobial therapy in a safety net hospital: opportunities for improvement. Open Forum Infect Dis 2024; 11:ofae190. 38778862 10.1093/ofid/ofae190 PMC11109603

[4] Norris AH, Shrestha NK, Allison GM, et al 2018 Infectious Diseases Society of America clinical practice guideline for the management of outpatient parenteral antimicrobial therapy. Clin Infect Dis 2019; 68:e1–35. 10.1093/cid/ciy745 30423035

[5] Petrak RM, Skorodin NC, Fliegelman RM, Hines DW, Chundi VV, Harting BP. Value and clinical impact of an infectious disease–supervised outpatient parenteral antibiotic therapy program. Open Forum Infect Dis 2016; 3:ofw193. 27807591 10.1093/ofid/ofw193 PMC5088696

[6] MacKenzie M, Rae N, Nathwani D. Outcomes from global adult outpatient parenteral antimicrobial therapy programmes: a review of the last decade. Int J Antimicrob Agents 2014; 43:7–16. 24200469 10.1016/j.ijantimicag.2013.09.006

[7] Berrevoets MAH, ten Oever J, Oerlemans AJM, Kullberg BJ, Hulscher ME, Schouten JA. Quality indicators for appropriate outpatient parenteral antimicrobial therapy in adults: a systematic review and RAND-modified Delphi procedure. Clin Infect Dis 2020; 70:1075–82. 31056690 10.1093/cid/ciz362 PMC7052541

[8] Reidy P, Breslin T, Muldoon E. Outpatient parenteral antimicrobial therapy (OPAT) across the world: a comparative analysis—what lessons can we learn? JAC Antimicrob Resist 2024; 6:dlae111. 39035018 10.1093/jacamr/dlae111 PMC11258576

[9] Herrera-Hidalgo L, Luque-Marquez R, de Alarcon A, et al Clinical outcomes of and innovative cefazolin delivery program for MSSA infections in OPAT. J Clin Med 2022; 11:1551. 35329878 10.3390/jcm11061551 PMC8950875

[10] Campbell PO, Gallagher K, Dalton SC, Metcalf SCL, Douglas NM, Chambers ST. Safety and clinical outcomes of outpatient parenteral antibiotic therapy for infective endocarditis in Christchurch, New Zealand: a retrospective cohort study. Int J Infect Dis 2023; 134:172–6. 37331565 10.1016/j.ijid.2023.06.008

[11] Azimi SF, Golnabi E, Mynatt RP, et al Antimicrobial stewardship practices in outpatient parenteral antimicrobial therapy programs in the United States. Open Forum Infect Dis 2024; 11:ofae347. 38983708 10.1093/ofid/ofae347 PMC11232693

[12] Mahoney MV, Childs-Kean LM, Khan P, Rivera CG, Stevens RW, Ryan KL. Recent updates in antimicrobial stewardship in outpatient parenteral antimicrobial therapy. Curr Infect Dis Rep 2021; 23:24. 34776793 10.1007/s11908-021-00766-x PMC8577634

[13] Burch AR, Ledergerber B, Ringer M, et al Improving antimicrobial treatment in terms of antimicrobial stewardship and health costs by an OPAT service. Infection 2024; 52:1367–76. 38421503 10.1007/s15010-024-02194-0 PMC11289230

[14] Kaul CM, Haller M, Yang J, et al Assessment of risk factors associated with outpatient parenteral antimicrobial therapy (OPAT) complications: a retrospective cohort study. Antimicrob Steward Healthc Epidemiol 2022; 2:e183. 36406163 10.1017/ash.2022.313 PMC9672913

[15] Means L, Bleasdale S, Sikka M, Gross AE. Predictors of hospital readmission in patients receiving outpatient parenteral antimicrobial therapy. Pharmacotherapy 2016; 36:934–9. 27393717 10.1002/phar.1799

[16] Mansour O, Heslin J, Townsend JL. Impact of the implementation of a nurse-managed outpatient parenteral antibiotic therapy (OPAT) system in Baltimore: a case study demonstrating cost savings and reduction in re-admission rates. J Antimicrob Chemother 2018; 73:3181–8. 30085088 10.1093/jac/dky294

[17] Barnes A, Nunez M. Diabetic foot infection and select comorbidities drive readmissions in outpatient parenteral antimicrobial therapy. Am J Med Sci 2021; 361:233–7. 33097196 10.1016/j.amjms.2020.08.027

[18] Epperson TM, Bennett KK, Kupiec KK, et al Impact of a pharmacist-managed outpatient parenteral antimicrobial therapy (OPAT) service on cost savings and clinical outcomes at an academic medical center. Antimicrob Steward Healthc Epidemiol 2023; 3:e15. 36714295 10.1017/ash.2022.374 PMC9879875

[19] Certain LK, Benefield RJ, Newman M, Zhang M, Thomas FO. A quality initiative to improve postdischarge care for patients on outpatient parenteral antimicrobial therapy. Open Forum Infect Dis 2022; 9:ofac19. 10.1093/ofid/ofac199 PMC9251666 35794930

[20] Peter S, Oberröhrmann C, Pfaff H, et al Exploring patients' perspectives: a mixed methods study on outpatient parenteral antimicrobial therapy (OPAT) experiences. BMC Health Serv Res 2024; 24:544. 38685017 10.1186/s12913-024-11017-9 PMC11057129

[21] Twiddy M, Czoski Murray CJ, Mason SJ, et al A qualitative study of patients' feedback about outpatient parenteral antimicrobial therapy (OPAT) services in Northern England: implications for service improvement. BMJ Open 2018; 8:e019099. 10.1136/bmjopen-2017-019099 PMC5781150 29326190

[22] Saillen L, Arendsdorff L, Moulin E, et al Patient satisfaction in an outpatient parenteral antimicrobial therapy (OPAT) unit pratising predominantly self-administration of antibiotics with elastomeric pumps. Eur J Clin Microbiol Infect Dis 2017; 36:1387–92. 28283831 10.1007/s10096-017-2944-5

[23] Wu KH, Sakoulas G, Geriak M. Vancomycin or daptomycin for outpatient antimicrobial therapy: does it make a difference in patient satisfaction? Open Forum Infect Dis 2021; 8:ofab418. 34476284 10.1093/ofid/ofab418 PMC8404740

[24] Hamad Y, Lane MA, Beekmann SE, Polgreen PM, Keller SC. Perspectives of United States–based infectious diseases physicians on outpatient parenteral antimicrobial therapy practice. Open Forum Infect Dis 2019; 6:ofz363. 31429872 10.1093/ofid/ofz363 PMC6765349

[25] Schranz AJ, Swartwood M, Ponder M, et al Quantifying the time to administer outpatient parenteral antimicrobial therapy: a missed opportunity to compensate for the value of infectious diseases. Clin Infect Dis 2024; 79:348–50. 38743581 10.1093/cid/ciae262 PMC11327780

[26] Gilchrist M, Barr D, Drummond F, et al Outpatient parenteral antimicrobial therapy (OPAT) in the UK: findings from the BSAC national outcomes registry (2015–19). J Antimicrob Chemother 2022; 77:1481–90. 35187565 10.1093/jac/dkac047

