# Supplementary material for: Outpatient Parenteral Antimicrobial Therapy Outcomes Metrics Assessment Survey
Source: Open Forum Infect Dis. 2025 May 12;12(5):ofaf283. doi: 10.1093/ofid/ofaf283 (PMC12117654; doi:10.1093/ofid/ofaf283)
Supplement: ofaf283_Supplementary_Data [file ofaf283_supplementary_data.docx]

| **Supplemental Table 1:** Survey outcome metrics scores | | | | | |
| --- | --- | --- | --- | --- | --- |
| **Survey Outcome Metric Scores** | | | | | |
| Outcome Metric | Perceived Importance Score  median (IQR)/SD | Currently collecting  n (%) | Collecting as part of a research or quality improvement project  n (%) | Attempted to collect but unable due to barriers  n (%) | Not collecting, never attempted  n (%) |
| Proportion of patients who are readmitted to the hospital during their OPAT course related to the infection or antimicrobial adverse effects | 10 (9-10) /1.45 | 33 (37.9) | 12 (13.8) | 7 (8.0) | 21 (24.1) |
| Clinical outcomes related to treatment of infections | 10 (8-10) /1.68 | 17 (19.5) | 16 (18.4) | 9 (10.3) | 30 (34.5) |
| Proportion of patients who have adverse reactions due to antimicrobials | 10 (8-10) /1.39 | 24 (27.6) | 16 (18.4) | 10 (11.5) | 23 (26.4) |
| Proportion of patients who complete their antibiotic course | 9 (8-10) / 1.88 | 21 (24.1) | 12 (13.8) | 9 (10.3) | 31 (35.6) |
| Proportion of patients who experience Peripherally Inserted Central Catheter (PICC) or other vascular access complications | 9 (8-10) /1.77 | 24 (27.6) | 16 (18.4) | 9 (10.3) | 24 (27.6) |
| Cost savings directly related to OPAT team interventions including reduction in ED visits, readmissions, and/or avoided line complications | 9 (7-10) /2.34 | 10 (11.5) | 13 (14.9) | 12 (13.8) | 37 (42.5) |
| Hospital days saved | 8 (7-10) /2.51 | 11 (12.6) | 10 (11.5) | 12 (13.8) | 39 (44.8) |
| Proportion of patients diagnosed with *Clostridioides difficile* infection during OPAT course | 8 (7-10) /2.24 | 16 (18.4) | 14 (16.1) | 6 (6.9) | 36 (41.4) |
| Rate of orals prescribing for complex infections | 8 (6-9) /2.56 | 4 (4.6) | 13 (14.9) | 8 (9.2) | 47 (54.0) |
| Patient satisfaction | 8 (6-9) /2.26 | 1 (1.1) | 8 (9.2) | 8 (9.2) | 55 (63.2) |
| Proportion of patients who require a change in antibiotics during the course | 8 (6-9) /2.04 | 19 (21.8) | 13 (14.9) | 12 (13.8) | 28 (32.2) |
| Antimicrobial selection | 8 (6-9) /1.97 | 11 (12.6) | 13 (14.9) | 7 (8.0) | 39 (44.8) |
| Time spent on various OPAT team interventions | 7 (6-10) /2.24 | 5 (5.7) | 14 (16.1) | 14 (16.1) | 39 (44.8 |
| Proportion of patients with missed labs | 7 (6-9) /2.18 | 10 (11.5) | 12 (13.8) | 13 (14.1) | 37 (42.5) |
| Length of hospital stay (LOS) prior to OPAT | 6 (5-8) /2.60 | 17 (19.5) | 10 (11.5) | 9 (10.3) | 36 (41.4) |
| Proportion of patient completing their OPAT course inpatient | 5 (3-8) /2.79 | 9 (10.3) | 8 (9.2) | 7 (8.0) | 46 (52.9) |
